# Forward‐Viewing Endoscopic Ultrasound‐Guided Fine‐Needle Biopsy for a Hypopharyngeal Carcinoma Mimicking a Subepithelial Lesion: A Case Report

**DOI:** 10.1002/deo2.70275

**Published:** 2026-01-19

**Authors:** Kakeru Otomo, Tadayuki Takagi, Jun Wada, Natsuki Ishizaki, Kana Tamazawa, Kohei Suzuki, Masato Aizawa, Hiroshi Ogawa, Osamu Suzuki, Kazutomo Togashi

**Affiliations:** ^1^ Department of Gastroenterology Aizu Medical Center Fukushima Medical University Aizuwakamatsu Japan; ^2^ Department of Gastroenterology Fukushima Medical University School of Medicine Fukushima Japan; ^3^ Department of Otolaryngology Aizu Medical Center Fukushima Medical University Aizuwakamatsu Japan; ^4^ Department of Diagnostic Pathology Aizu Medical Center Fukushima Medical University Aizuwakamatsu Japan

**Keywords:** EUS‐FNA, EUS‐FNB, EUS‐TA, forward‐viewing curved linear‐array echoendoscope (FV‐EUS), head and neck tumor

## Abstract

Subepithelial lesions (SELs) of the head and neck have a low diagnostic yield with mucosal biopsy and carry a bleeding risk. Among endoscopic ultrasound–tissue acquisition (EUS‐TA) techniques, fine‐needle biopsy (FNB) provides higher specimen adequacy and diagnostic accuracy than fine‐needle aspiration (FNA). A forward‐viewing curved linear‐array echoendoscope (FV‐EUS) is useful for mobile lesions and those in narrow spaces that are difficult to puncture with conventional oblique‐viewing EUS (OV‐EUS), but FV‐EUS–guided EUS‐FNB has not been reported for head and neck lesions.

We report a 72‐year‐old man in whom a lesion at the esophageal inlet was not apparent on initial upper gastrointestinal endoscopy performed with a small‐caliber endoscope. Stenosis was subsequently noted at the time of endoscopic submucosal dissection for early gastric cancer. Contrast‐enhanced computed tomography showed an approximately 20‐mm solid mass on the posterior hypopharyngeal wall. Under general anesthesia with laryngoscopic exposure, an elevated subepithelial lesion without mucosal exposure was observed on the posterior pharyngeal wall. Using FV‐EUS with a cap device attached to the scope tip, a 22 × 18 mm hypoechoic subepithelial mass was clearly visualized, and consecutive EUS‐FNB was performed with a 22‐gauge needle. No complications, including bleeding, occurred. Histopathology and immunohistochemistry demonstrated moderately differentiated squamous cell carcinoma, and the patient was referred for treatment as primary hypopharyngeal cancer. This case illustrates the feasibility and safety of FV‐EUS–guided FNB for pharyngeal SELs and suggests a wider role for FV‐EUS in head and neck disorders.

## 1 Introduction

Endoscopic ultrasound‐fine‐needle aspiration (EUS‐FNA) is widely used to obtain pathological specimens from gastrointestinal subepithelial lesions (SELs), but its diagnostic accuracy can be lower than that for pancreatic masses because of puncture difficulty and the frequent need for immunohistochemical (IHC) evaluation. Forward‐viewing echoendoscopes and rapid on‐site evaluation (ROSE) have been introduced to improve tissue acquisition.

In the head and neck region, bite‐on‐bite biopsy and mucosal incision–assisted biopsy (MIAB) are commonly used. However, the diagnostic yield of bite‐on‐bite biopsy for SELs is 38%, and bleeding requiring hemostasis has been reported in 13.5% of cases [1]. Although MIAB achieves a high diagnostic rate, it requires incision, dissection, and closure, and bleeding control remains challenging [2].

Endobronchial ultrasound‐guided transbronchial needle aspiration (EBUS‐TBNA) is another option that is safe and highly diagnostic for lung cancer and mediastinal lymph‐node metastasis [3, 4], but reports in pharyngeal tumors or gastrointestinal SELs are limited, and EBUS‐TBNA alone may fail to obtain material adequate for IHC [5].

EUS‐tissue acquisition (EUS‐TA) allows real‐time puncture while visualizing wall layers and vessels. Meta‐analyses have shown that fine‐needle biopsy (FNB) yields higher tissue adequacy, core procurement, and overall diagnostic accuracy than FNA. The forward‐viewing design of the curved linear‐array echoendoscope (FV‐EUS) enables coaxial advancement of devices and stable puncture, and cap‐assisted fixation facilitates sampling of small or mobile lesions. FV‐EUS has been reported to be useful for lesions that are difficult to puncture with conventional oblique‐viewing EUS (OV‐EUS) and to provide larger tissue samples [6, 7, 8, 9]. To our knowledge, EUS‐FNB using FV‐EUS has not previously been described for lesions in the head and neck region. We report such a case and discuss technical and anatomical considerations.

## 2 Case Presentation

**FIGURE 1:**
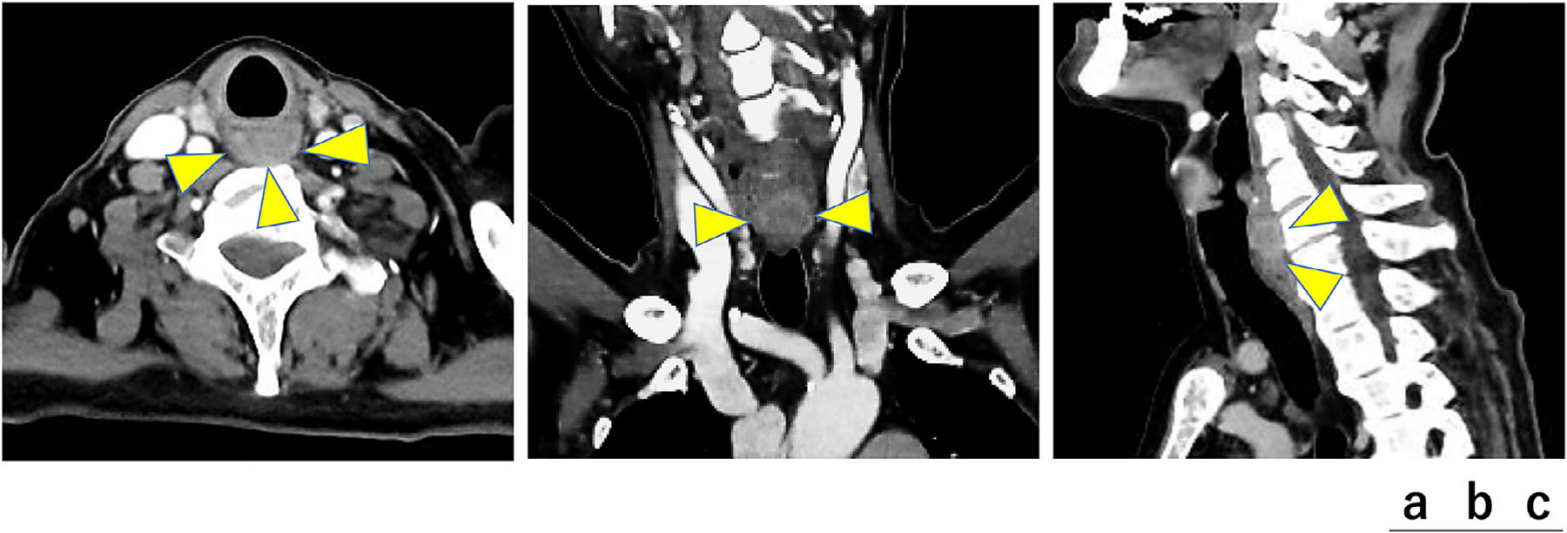
Contrast‐enhanced computed tomography (CT) of the head and neck. (a) Axial image. An approximately 20 mm mass with rim enhancement and relatively poor internal enhancement is seen posterior to the main bronchus (arrowhead). (b) Coronal image. A mass with rim enhancement and relatively poor internal enhancement is seen (arrowhead). (c) Sagittal image. At the level of the inferior border of the cricoid cartilage and ventral to the sixth cervical vertebra, a mass with rim enhancement and relatively poor internal enhancement is noted.

**FIGURE 2:**
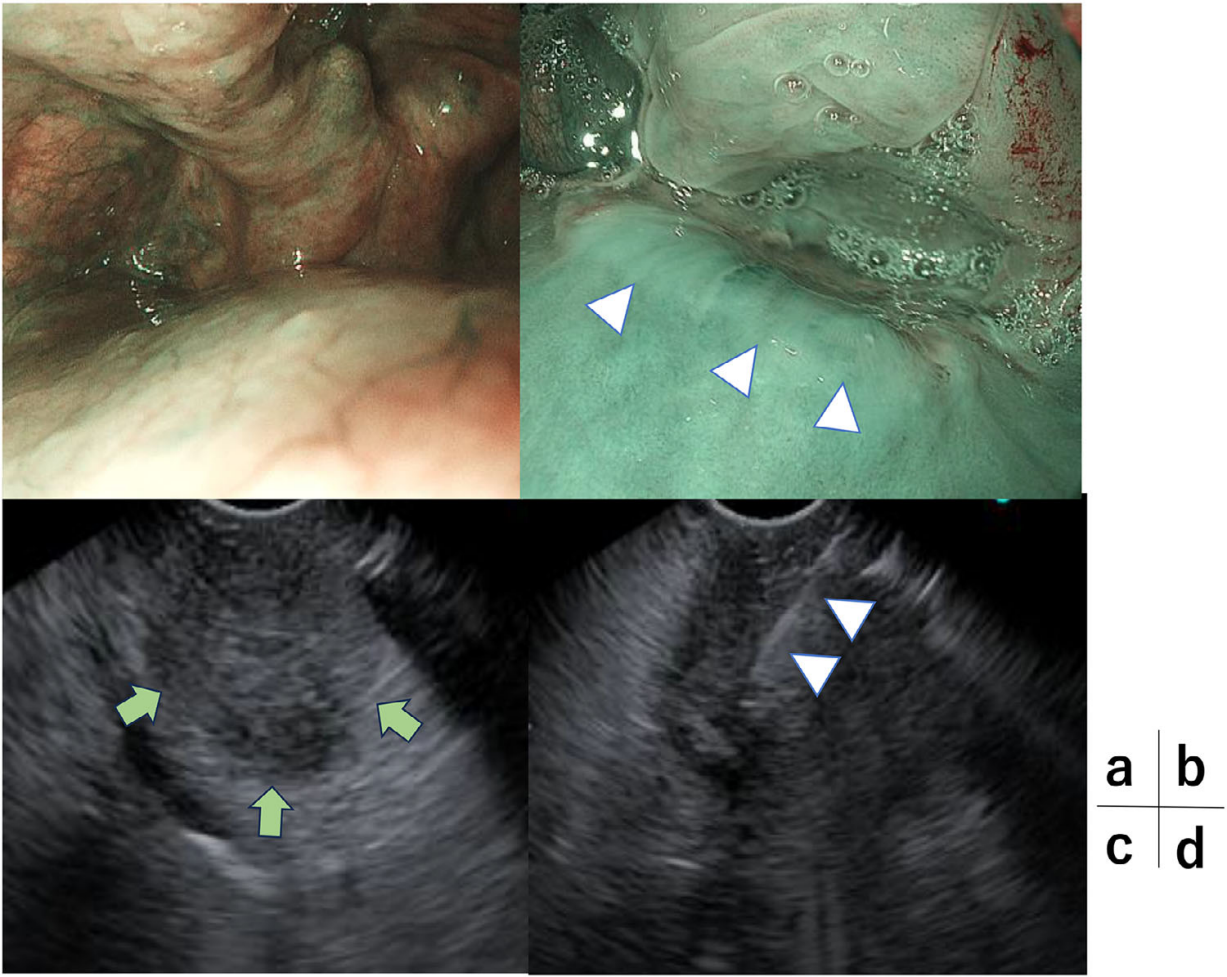
Endoscopic views. (a) Narrow‐band imaging (NBI) close‐up without laryngoscopic exposure (GIF‐H290T); the lesion is difficult to visualize. (b) NBI close‐up after laryngoscopic exposure (GIF‐XZ1200) showing a subepithelial‐like bulge with slight central depression on the posterior hypopharyngeal wall without mucosal exposure (arrowhead). (c) Endoscopic ultrasound (EUS) image (TGF‐UC260J) demonstrating a 22 × 18 mm, relatively well‐demarcated, ovoid hypoechoic subepithelial lesion (arrow). Internal echogenicity appears relatively homogeneous. (d) EUS‐fine needle biopsy (EUS‐FNB) image showing needle puncture with an Acquire S 22‐gauge needle under EUS guidance (arrowhead).

A 72‐year‐old man presented with pharyngeal discomfort. Esophagogastroduodenoscopy for iron‐deficiency anemia revealed early gastric cancer, and he was referred to our department. The initial upper gastrointestinal endoscopy with a small‐caliber endoscope did not reveal any obvious lesion at the esophageal inlet. At the time of endoscopic submucosal dissection (ESD) for early gastric cancer, stenosis was noted in the left hypopharynx. Passage of a standard upper gastrointestinal endoscope (GIF‐H290T) was barely possible, and detailed observation was difficult. Contrast‐enhanced computed tomography showed an approximately 20‐mm solid mass on the posterior hypopharyngeal wall (Figure 1). Because severe stenosis was expected to hamper insertion and observation with EUS, we planned the procedure under general anesthesia with laryngoscopic exposure in collaboration with otolaryngologists. After induction of anesthesia, laryngeal exposure was obtained using Sato's curved laryngoscope, and a high‐magnification endoscope (GIF‐XZ1200) was advanced. A subepithelial‐like bulge with slight central depression on the posterior hypopharyngeal wall without mucosal exposure was observed, and FV‐EUS (TGF‐UC260J) with an attached cap device demonstrated a 22 × 18 mm hypoechoic subepithelial mass (Figure 2).

**FIGURE 3:**
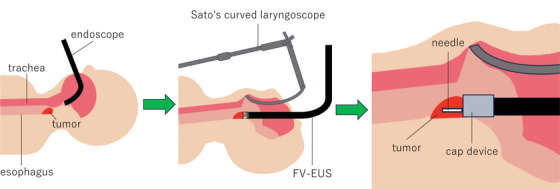
A forward‐viewing curved linear‐array echoendoscope (FV‐EUS)‐guided fine needle biopsy (FNB) with Sato's curved laryngoscope.

Using a 22‐gauge Franseen‐tip FNB needle (Acquire S), EUS‐FNB was performed under EUS guidance. Negative pressure with a 20‐mL syringe was applied, and two passes with 20 to‐and‐fro movements were made. During sampling, the lesion was sucked into the attached cap, allowing fixation by cap suction and coaxial needle advancement. ROSE showed atypical epithelial cells, and frozen‐section rapid pathology diagnosed squamous cell carcinoma. No complications, including bleeding at the puncture site, occurred, and the procedure was completed (Figure 3 and Video S1).

The postoperative course was uneventful, and the patient was discharged on postoperative day 4. Histological examination of the permanent specimen revealed sheet‐like proliferation of atypical epithelial cells with keratinization and intercellular bridges. Immunohistochemistry showed positivity for AE1/AE3 and p40 with an increased MIB‐1 (Ki‐67) labeling index, whereas chromogranin A, synaptophysin, CD56, HMB45, MelanA, and S‐100 were negative, confirming moderately differentiated squamous cell carcinoma. No other lesions were found on further evaluation, and the lesion was regarded as primary hypopharyngeal cancer. The patient was referred to another hospital for definitive treatment.

## 3 Discussion

Although EUS‐FNB is now recommended as a first‐line approach for gastrointestinal SELs, its use in the head and neck region remains limited. This case shows that FV‐EUS–guided FNB can provide a rapid and safe definitive diagnosis for a subepithelial‐like lesion in this area. Bite‐on‐bite biopsy for SELs has a diagnostic yield of 38%, and bleeding requiring hemostasis occurs in 13.5% of cases [1]. MIAB offers a higher diagnostic rate, particularly for lesions ≤20 mm, but requires incision, dissection, and closure, and bleeding control is still problematic [2].

EBUS‐TBNA is the gold standard for diagnosing lung cancer and mediastinal lymph‐node metastasis, with high diagnostic performance and safety [3, 4]. However, its role in pharyngeal tumors and gastrointestinal SELs has not been fully established, and cases have been reported in which EBUS‐TBNA alone did not provide specimens adequate for IHC in esophageal SELs [5]. Although FNB needles are now available for EBUS‐TBNA, a trans‐airway approach imposes limitations in puncture distance and angle for lesions such as those of the hypopharynx or posterior pharyngeal wall. For such lesions, a transesophageal approach with FV‐EUS may therefore be more suitable.

FV‐EUS provides forward‐viewing optics and a working channel aligned with the endoscope shaft, enabling coaxial and straight puncture from the front of the lesion. In combination with cap‐assisted suction, this facilitates stable targeting and tissue acquisition even in small or mobile lesions. Overall diagnostic rates are comparable between FV‐EUS and OV‐EUS, but FV‐EUS has been reported to obtain larger tissue samples and to shorten procedure times [6, 7, 8]. FNB further improves specimen adequacy and diagnostic accuracy compared with FNA, with fewer required passes [9]. In the present case, OV‐EUS was expected to be difficult to advance because of stenosis. The shorter rigid tip and forward ultrasound viewing direction of FV‐EUS allowed visualization just proximal to the stenosis, consistent with previous reports describing its usefulness when OV‐EUS access is limited [10]. Adequate specimens were obtained with only two passes, suggesting that this approach may be applicable not only to malignant tumors but also to a variety of pharyngeal SELs.

Because stenosis was severe, general anesthesia with Sato's curved laryngoscope exposure was required. Completing diagnosis in a single session reduces patient burden, and the combination of ROSE and intraoperative rapid histology was therefore appropriate. The relatively large outer diameter of FV‐EUS increases the risk of contact with the laryngoscope and endotracheal tube; pre‐procedural planning with otolaryngologists and anesthesiologists regarding fixation and positioning is essential. At our institution, to reduce the risk of complications such as tongue edema, dysgeusia, and pharyngodynia, the continuous suspension time with the laryngoscope is limited to within 30 min and the total suspension time within 60 min, and the present examination was performed safely without adverse events under these conditions.

In summary, FV‐EUS–guided FNB is a safe and effective option for qualitative diagnosis of subepithelial lesions in the head and neck region. It is less invasive than conventional surgical or mucosal incision–based methods and may be particularly useful when a single‐session diagnosis under general anesthesia is desired. Further accumulation of cases is needed to define optimal indications and clarify the diagnostic performance of this technique.

## Author Contributions

**Investigation**: Kakeru Otomo; **Supervision**: Tadayuki Takagi and Kazutomo Togashi; **Writing – original draft preparation**: Kakeru Otomo; **Writing – review and editing**: all authors; **Approval of the final version of the manuscript**: all authors.

## Conflicts of Interest

The authors declare no conflicts of interest.

## Funding

The authors did not receive any specific funding for this work.

## Supporting information




**Supporting Video 1**: Intraoperative procedure showing FV‐EUS‐guided fine‐needle biopsy under general anesthesia.
